# Getting the Joke: Insight during Humor Comprehension – Evidence from an fMRI Study

**DOI:** 10.3389/fpsyg.2017.01835

**Published:** 2017-10-18

**Authors:** Fang Tian, Yuling Hou, Wenfeng Zhu, Arne Dietrich, Qinglin Zhang, Wenjing Yang, Qunlin Chen, Jiangzhou Sun, Qiu Jiang, Guikang Cao

**Affiliations:** ^1^Key Laboratory of Cognition and Personality (SWU), Ministry of Education, Chongqing, China; ^2^Faculty of Psychology, Southwest University, Chongqing, China; ^3^Department of Psychology, American University of Beirut, Beirut, Lebanon

**Keywords:** insight, humor comprehension, incongruity-resolution theory, character, language-free cartoon, functional magnetic resonance imaging (fMRI)

## Abstract

As a high-level cognitive activity, humor comprehension requires incongruity detection and incongruity resolution, which then elicits an insight moment. The purpose of the study was to explore the neural basis of humor comprehension, particularly the moment of insight, by using both characters and language-free cartoons in a functional magnetic resonance imaging study. The results showed that insight involving jokes elicited greater activation in language and semantic-related brain regions as well as a variety of additional regions, such as the superior frontal gyrus (SFG), the inferior frontal gyrus (IFG), the middle temporal gyrus (MTG), the superior temporal gyrus (STG), the temporoparietal junctions (TPJ), the hippocampus and visual areas. These findings indicate that the MTG might play a role in incongruity detection, while the SFG, IFG and the TPJ might be involved in incongruity detection. The passive insight event elicited by jokes appears to be mediated by a limited number of brain areas. Our study showed that the brain regions associated with humor comprehension were not affected by the type of stimuli and that humor and insight shared common brain areas. These results indicate that one experiences a feeling of insight during humor comprehension, which contributes to the understanding of humor comprehension.

## Introduction

As an important high-level cognitive activity, humor plays a crucial role in human social life. Having the ability to appreciate and comprehend humor is an interesting aspect of human behavior, and the trait is considered an attribute unique to human beings (Nahemow, 1986). Suls (1972) proposed incongruity-resolution theory and suggested that the cognitive processing aspects of a joke (humor) could be divided into two stages: incongruity detection and incongruity resolution. In humor comprehension, incongruity means that two or more incompatible schemas are activated in the same situation simultaneously, and incongruity detection means that subjects notice the existence of incompatible schemas, that is, the uncertainty of selective activation of multiple schemas in a given concept. In addition, the process of extracting appropriate schema from multiple schemas according to the current situation is incongruity resolution (Wyer and Collins, 1992). Thus, the process of humor comprehension is processed in a step-by-step procedure (Coulson, 2001).

In incongruity detection, uncertainty may lead to surprise, which consists of a series of immediate reactions, such as cognition interruption, attention assignments, and more systematic handling of surprising things (Meyer et al., 1997; Topolinski and Strack, 2015). Surprise can interrupt ongoing activities and thinking patterns, requiring an increase in processing depth to cognitively master unexpected events (surprising stimulus) (Meyer et al., 1997; Topolinski and Strack, 2015). Such interruptions tend to cause negative feelings (Noordewier and Breugelmans, 2013) because uncertainty means that one has failed to predict future events (Huron, 2006). However, in incongruity resolution, people can feel pleasant once the surprising outcome is understood (Noordewier et al., 2016). That is, the punchline of a joke elicits a moment of insight, and the ease of this insight can make people feel funnier and experience more enjoyment (Topolinski, 2014).

Bekinschtein et al. (2011) claimed that jokes appear to involve executive functions, such as thought organizing, insight development, information disambiguating, schema shifting and bridging inferences to re-establish a new context. To ensure a joke works well, the first part of the joke (incongruity detection) creates a context (C1), which can induce the subject to assume a hypothesis (H1). The subjects may formulate several hypotheses because the joke is relatively ambiguous. It is necessary for the subjects to go back and reprocess the first part of the joke to find an alternative explanation, which leads to the second hypothesis (H2) (Jodłowiec, 1991). The punchline of a joke induces an insight that comes from the re-comprehension or reinterpretation of the context and the problem. Thus, humor comprehension could be regarded as a problem-solving task (Suls, 1972). When the problem or the incongruity is resolved, the old frame will be shifted to a new one (Oakley and Coulson, 2000). Subjects gain a new perspective for perceiving the problem and understand it; thus, the new perspective leads to a feeling of insight.

Several previous imaging studies have examined the neural basis of humor comprehension using two types of materials: visual materials (cartoons, visual puns and short movie clips or verbal materials (phonological and semantic) (Azim et al., 2005; Bartolo et al., 2006; Bekinschtein et al., 2011; Chan et al., 2012, 2013; Amir et al., 2013). In comparing non-funny or nonsense conditions with funny visual stimulus conditions, the activated regions observed under funny visual conditions included the middle temporal gyrus (MTG), inferior frontal gyrus (IFG), superior frontal gyrus (SFG), middle frontal gyrus (MFG), and the temporoparietal junctions (TPJ) during humor comprehension (Azim et al., 2005; Bartolo et al., 2006; Kohn et al., 2011; Marinkovic et al., 2011; Neely et al., 2012). For verbal jokes, the main activated regions have been detected in the MTG, ITG, SFG, IFG, and TPJ (Bekinschtein et al., 2011; Chan et al., 2012). Several common regions under visual and verbal conditions include the TPJ, MTG, ITG, IFG, SFG, and MFG (Vrticka et al., 2013). However, only one type of experimental material (visual material or verbal material) has been used in previous studies about humor; therefore, it is difficult to compare the brain regions associated with different conditions in a study.

Gestalt theorists note that the reconstruction of certain changes during humor comprehension may lead to a higher level of cognition (DERKS, 1987; Gick and Lockhart, 1995) and that the cognitive processes involved in humor comprehension likely share certain features with those involved in insight (Gick and Lockhart, 1995). Insight occurs in a particular problem situation, and there is no inner speech at the critical moment (Schooler et al., 1993; Gick and Lockhart, 1995). Reconstruction, the shift in problem representation, is the essential characteristic of insight. Ohlsson (1984) indicated that reconstruction occurs during problem solving, but reconstruction does not necessarily promote problem solving. In addition, it is more likely to be the depth analysis of problems and the goals that often must break from the process of chunk decomposition. Chunks consist of different types of elements and are gradually formed in people’s daily life. Whether a problem representation can be effectively converted depends on the proximity of the elements in the associated chunk. As such, an insight experience may lead to perception, problem solving, language comprehension and other domains of cognition (Ohlsson, 1992; Cunningham et al., 2009). Amir et al. (2013) conducted an functional magnetic resonance imaging (fMRI) study to explore the neural differences between humor comprehension and insight. The results indicated that the brain regions associated with insight interpretation overlapped with the regions related to humor interpretation. Therefore, the study of insight during humor comprehension could contribute to the understanding of insight and humor.

Several previous studies have examined the neural basis of insight using traditional problem-solving tasks. The results have shown increased activation in the superior temporal gyrus (STG), prefrontal cortex (PFC), cingulate cortex (ACC), hippocampus and temporoparietal cortices (Luo and Niki, 2003; Bechtereva et al., 2004; Luo et al., 2004; Qiu et al., 2008a,b; Aziz-Zadeh et al., 2009; Dietrich and Kanso, 2010). In a study of verbal tasks, many types of problems, such as riddles, anagrams, the Remote Associates Test (RAT) and other forms of insight problems, were used as verbal materials (Mednick, 1962). The RAT was developed by Mednick (1962) and has since been considered a valid tool for measuring creativity. Each RAT question presents three cued words that are linked by a fourth word, which is the correct answer (for the triad “athletes, web and rabbit,” the answer is “food”). Many spatial insight problems, such as the 4-dot problem, the figure problem and the pennies problem, have been used as visual materials in the study of visual tasks. The experimental paradigm and conclusions of these studies have been criticized (Dietrich and Kanso, 2010; Weisberg, 2013). The reasons for this phenomenon are as follows. First, the brain regions associated with insight appeared to be more diverse. Second, a large number of different types of insight problems were used in these studies, but some did not meet the criteria of insight problems (Dietrich and Kanso, 2010).

To date, many studies have studied the neural basis of humor comprehension. However, only a single type of material has been used in those studies, such as visual materials (cartoons and visual puns) or verbal materials (phonological and semantic), without directly comparing visual materials vs. verbal materials. Although humor and insight have much in common in cognitive processing and neural mechanisms, previous studies have addressed these topics separately; indeed, no study has addressed both humor and insight. Therefore, using two different types of materials represents an improvement over current research. In the present study, our aim was to explore the neural basis of humor comprehension using two types of materials including a character condition (verbal) and a language-free cartoon condition (visual). In particular, our objective was to examine whether the brain regions activated in humor comprehension were affected by the different materials. In addition, we set out to study whether the punchline of a joke can elicit a moment of insight during humor comprehension. We speculated that, first, there might be overlaps in the activated brain regions under different conditions because previous studies of humor (using verbal materials or visual materials) found overlapping brain regions; second, we speculated that the participants might have a feeling of insight when they read the jokes and that humor shares some brain regions in common with insight.

## Materials and Methods

### Subjects

As paid volunteers, 33 participants (17 females, 16 males) aged 18–25 years (mean age, 21.03 years) from Southwest University in China were involved in the experiment. Nine participants were excluded because of head motion > 3 mm maximum translation or 3° rotation during fMRI scanning. The final sample consisted of 24 participants (11 females, 13 males) aged 19–25 years (mean age, 21.13 years). All participants were native Chinese speakers who had normal or corrected-to-normal vision and reported no present or past neurological or psychiatric disorders. The experiment was approved by the Academic Committee of the School of Psychology and the local ethics committee of the School of Psychology, Southwest University in China. All participants signed informed consent forms before participating in the study.

### Design and Materials

**FIGURE 1:**
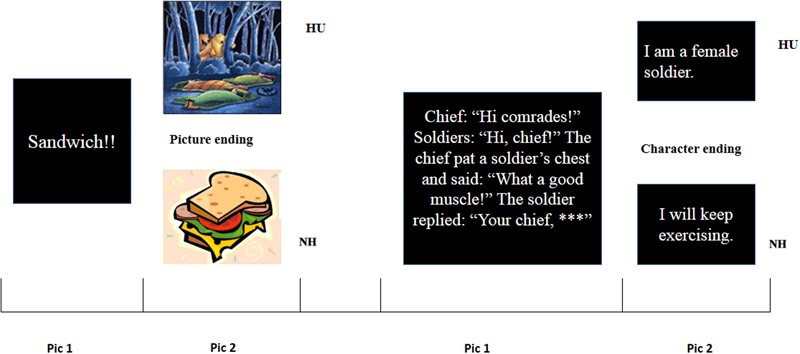
Materials.

A 2 (ending presentation: pure character vs. pure picture) × 2 [conditions: humorous (HU) vs. non-humorous (NH)] within-subjects design was used. Two pictures made up a story. Picture 1 (pic 1) was the situation background (setup) of the story, and picture 2 (pic 2) was the ending of the story. The pictures were shown in **Figure 1**. For each group, half were in the HU condition and the rest were in the NH condition.

Prior to the experiment, 220 jokes were selected from the Internet, books and previous research (Tu et al., 2014). Half of the joke endings were presented purely in characters, and the other half were presented purely in pictures. All jokes had two parts. The first part was the situation background; the second part was the ending, which included the punchline. Whether a joke was humorous mainly depended on its surprising ending and the punchline. All collected stories were humorous jokes. However, to ensure the consistency of the situation background, we prepared a corresponding non-humorous ending for each joke; that is, the first part of every joke did not change, and the second part had one of two endings (humorous or non-humorous). For example, the first part of the story (situation background) was “A man wants to buy a videotape at a store and the salesman asks him if he want to buy the light music. He says that both the light and the weight are OK.” The humorous ending was “I drive my car,” and the non-humorous ending was “I love it.” The other 23 subjects (13 females, 10 males), aged 18–24 years (mean age, 20.79 years), did not join the fMRI experiment and were similar in age and education background. We asked these subjects to rate each story on a scale of 1 to 4 (1 = incomprehension, 2 = non-humorous, 3 = a little humorous, 4 = humorous). We analyzed the data with SPSS. We also referred to the principles used in a previous study to select the materials (Tu et al., 2014). The stories rated more than 16 times were considered humorous, and the stories rated fewer than 3 times were considered non-humorous. Ultimately, 60 stories (HU vs. NH) comprised our experiment materials, including 30 stories involving only pictures and 30 stories involving only characters. We checked materials several times to ensure that there were no grammatical errors in the text of these stories. In addition, sentence length and the familiarity of the words were matched because we believed that if the length and familiarity were not appropriate, the credibility of the experimental data would be reduced. All these processes were designed to ensure the quality of the experimental data collected in the following fMRI experiment.

We expected the reliability and validity of the experimental materials to be high. More specifically, the majority of the HU trials were rated as humorous and surprising by participants, and the majority of the NH trials were rated as non-humorous and non-surprising by participants.

### Procedures

**FIGURE 2:**
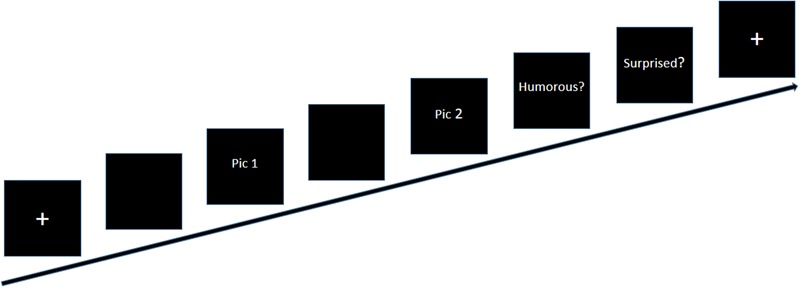
A flow chart of stimulus presentation in each trial.

To make the participants familiar with the task, all participants were asked to complete a brief set of trials in each condition. They were asked not to move their heads during the scanning; imaging data were then recorded. A flow chart of the formal experiment is shown in **Figure 2**. First, a white fixation point (+) appeared in the center of the black screen; the black screen remained once the white fixation point disappeared. Then, the first part of the story (pic 1), that is, the situation background, was presented, and the participants were asked to press the “1” button immediately once they understood it; however, pic 1 did not disappear until the presentation time (8, 10, or 12 s), which was set according to the results of a pilot study. Second, the second screen of the story (pic 2) followed the first screen. Participants were again instructed to press the “1” button once they understood the story. The picture did not disappear until the presentation time (6, 8, or 10 s). Finally, two evaluation questions were presented. The evaluation questions did not disappear when the participants gave a response, and the question was displayed for a fixed duration of 4 s. The questions were as follows: “Do you think the story is humorous?” (1 = incomprehension, 2 = non-humorous, 3 = a little humorous, 4 = humorous) and “Do you think the story is surprising?” (1 = not surprising, 2 = a little surprising, 3 = surprising). A fixation point (+) signaled the next trial. The presentation time of the fixation point (+) and the blank black screen were randomly set within the range 0.5 s ∼ 4.5 s.

All stories were divided into 6 blocks, each of which included 20 trials; thus, there were 120 trials in total. In the first 3 blocks, pic 2 was presented with only images, while in the latter 3 blocks pic 2 was presented with only characters. Each trial was presented in a pseudo-random order and only once in each block. To ensure the quality of the behavioral data and brain imaging data, we asked each participant to answer four questions after the scanning sessions. The first two questions were as follows: “Were you in a good mental state when you lay in the scanner?” and “Does lying in the instrument affect your judgement?” We also asked the participants “Did you have a feeling of insight when you understood the ending of the story?” We believe the question was consistent with the purpose of our experiment and could help us determine whether the participant experienced insight during humor comprehension. Finally, to collect information about the frequency with which each participant read jokes in their daily life, we asked the following question: “Do you often read jokes?” The purpose of asking these questions was to ensure the quality of the experimental data for the fMRI data that we acquired because the quality the brain imaging data and the performance in the experiment would be effected by the mental state of the participant.

### fMRI Data Acquisition

All images were collected with a Siemens 3T scanner (Siemens Magnetom Trio TIM, Erlangen, Germany). Head movement was minimized by restraining the participant’s head using a vacuum cushion. Participants were also instructed to keep still. A screen was located at the rear of the scanner, and the participants could see the stimulus displayed on the screen through a mirror mounted on the standard head coil. In all sessions, to eliminate the magnetic saturation effect, the first five time points were removed.

BOLD images were obtained using an Echo Planar Imaging (EPI) sequence [slices = 32, voxel size = 3.4 mm × 3.4 mm × 4 mm; TR = 2000 ms; TE = 30 ms; field of view (FOV) = 200 mm × 200 mm; flip angle = 90°; thickness = 3 mm; slice gap = 1 mm]. T1-weighted high resolution anatomical images were collected for each participant (slices = 176; voxel size = 1 mm × 1 mm × 1 mm; TR = 1,900 ms; TE = 2.52 ms; FOV = 256 mm × 256 mm; flip angle = 90°; thickness = 1 mm).

### fMRI Data Analysis

We analyzed brain imaging data with SPM8 software^1^ (Welcome Department of Imaging Neuroscience, London, United Kingdom). First, functional images were corrected for the slice acquisition time of each volume and by rearranging the first volume to correct for head motion. Participants who exhibited head motion exceeding 3 mm of maximum translation or 3.0° of rotation were excluded. Then, these images were normalized to the MNI EPI template (voxel size, 3 mm × 3 mm × 3 mm). The normalized data were spatially smoothed with a Gaussian kernel, and the full width at half-maximum (FWHM) was specified as 8 mm × 8 mm × 8 mm. A high-pass filter was implemented with a cut-off period of 128 s to remove low-frequency drift from the time series.

After pre-processing, the data for each participant were analyzed with the general linear model (GLM). The movement correction parameters were added as covariance of no interest. Using a canonical haemodynamic response function, the BOLD signal was modeled by convolving the design matrix. The design matrix contained six sessions. Each session consisted of three conditions: NH, HU, and Fix. We analyzed the trials in which the participants chose the appropriate response and the inappropriate response (humorous for the HU condition and non-humorous for the NH condition, and Fix referred to the screen of the fixation point). The onsets of these three conditions for each participant were modeled, and each trial was treated as an independent event. We analyzed the time window spanning from the beginning of the presentation of the second screen (picture 2) to the response made by the participant and the BOLD signal during this period. In addition, six realignment parameters for each participant were modeled as confounding factors.

Next, a second-level analysis was performed, which included 24 participants. The first-level analysis of each participant produced three contrast images (NH, HU, and Fix) related to each condition modeled. The results of the first-level analysis were analyzed using a paired *t*-test to estimate the different activations between HU and HU. For all the analyses, the threshold was set to *p* < 0.05 (FDR corrected) cluster sizes = 100. FDR (false discovery rate) correction was performed at the voxel level. FDR did not control the type I error rate (Finner and Roters, 2001).

## Results

### Behavioral Results

Approximately 79.0 ± 12.7% of the HU trials were rated humorous and surprising by the participants, and approximately 70.8 ± 12.3% of the NH trials were rated non-humorous and no-surprise by the participants. The scores pertaining to humor and surprise for the subjects in the HU and NH conditions are shown in **Table 1**. The mean scores for humor in the HU and NH conditions were significantly different [*t*(23) = 28.11, *p* < 0.0001, 1-β > 0.8], and the mean scores for surprise in the HU and NH conditions were significantly different [*t*(23) = 12.65, *p* < 0.0001, 1-β > 0.8].

**Table 1:** The mean and standard deviation of humorous and surprise scores.

	Ending	Number	Mean	*SD*
Humor	Humorous ending	24	3.449	0.252
	Non-Humorous ending	24	2.000	0.010
Surprise	Surprise ending	24	2.278	0.326
	No-Surprise ending	24	1.284	0.205

We also analyzed the post-scan questions, and the results were as follows: 95% of participants said that they were in a good mental state during the scanning and that their judgements were not affected by the scanner. Moreover, 75% of participants said that they experienced insight when they read the jokes. The result was consistent with our expectation that there is a moment of insight during humor comprehension. Furthermore, 62.5% of the participants reported that they often read jokes. This result provided a reference value for this study, but we do not discuss this result here in because our goal was to collect information about the frequency with which the participants read jokes in their daily life.

### Imaging Data

#### Whole-Brain Analysis

**FIGURE 3:**
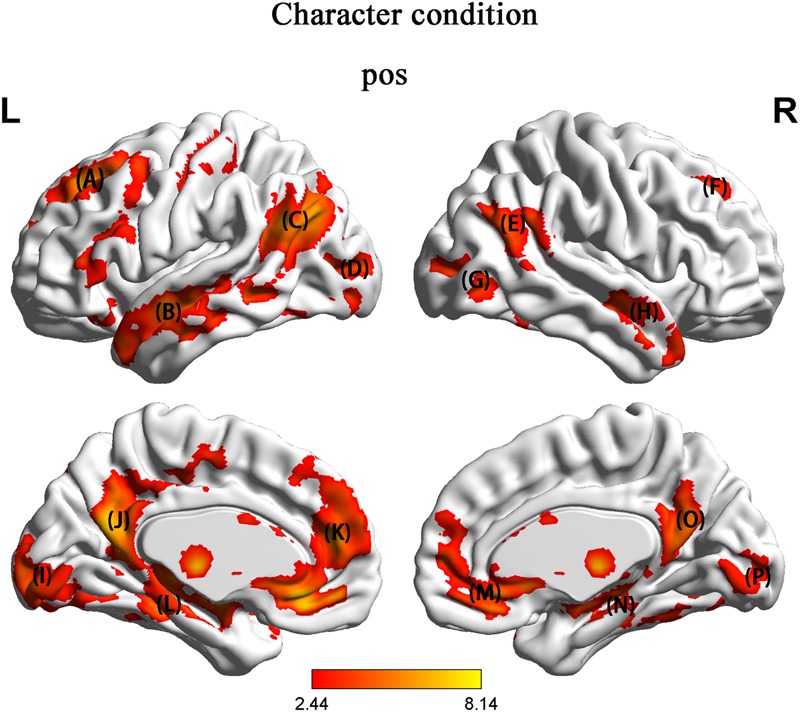
In contrast to NH, HU showed increased activation in region such as the (A) left superior frontal gyrus (SFG) and medial orbital frontal gyrus (MFO) and inferior frontal gyrus (IFG) (B) left middle temporal gyrus (MTG) (C) left temporo-parietal junction (TPJ) (D) left superior occipital gyrus (SOG) (E) right superior temporal gyrus (STG) (F) right middle frontal gyrus (MFG) (G) right superior occipital gyrus (SOG) (H) right middle temporal gyrus (MTG) (I) left middle occipital gyrus (MOG) (J) posterior cingulate cortex (PCC) (K) the prefrontal cortex (PFC) (L) hippocampus (M) subgenual anterior cingulate cortex (sgACC) (N) hippocampus (O) posterior cingulate cortex (PCC) (P) right middle occipital gyrus (MOG).

We focused on brain activation during the presentation of pic 2. We tested brain activity by contrasting HU with NH. In the character condition, in contrast to NH, HU showed increased activation in regions such as the left SFG, right MFG, left IFG, bilateral MTG, right STG, left TPJ, bilateral MOG, left precentral, posterior cingulate cortex (PCC), subgenual anterior cingulate cortex (sgACC) and hippocampus (**Figure 3** and **Table 2**).

**Table 2:** Regions of significant activation *p* < 0.05 (FDR corrected).

Brain regions	BA	MNI coordinates			*t*-value	Cluster size
		*x*	*y*	*z*		
**Character (HU > NH)**						
R. Middle temporal gyrus		57	-9	-12	4.40	343
L. Middle temporal gyrus		-57	-3	-12	5.87	806
R. Superior temporal gyrus		57	-51	21	5.04	500
R. Middle frontal gyrus		24	27	42	5.95	183
L. Superior frontal gyrus		-24	42	42	3.90	327
L. Temporo-parietal junction (TPJ)		-51	-60	27	6.59	257
L. Precentral		-39	-18	51	6.35	594
L. Middle occipital gyrus		-27	-84	3	3.46	534
R. Middle occipital gyrus	19	42	-75	3	3.87	138
Posterior cingulate cortex		-6	-57	18	6.07	309
Subgenual anterior cingulate cortex		3	27	-9	4.94	313
hippocampus		-24	-12	-12	5.48	201
**Picture (HU > NH)**						
L. Superior temporal gyrus		-60	0	-12	6.63	212
L. Middle temporal gyrus	39	-45	-63	18	6.00	695
R. Middle temporal gyrus		54	-51	15	5.99	337
L. Triangle inferior frontal gyrus		-54	39	12	4.68	116
L. Superior frontal gyrus		-9	51	33	4.90	114
L. Fusiform		-27	-36	-21	4.78	122
R. ParaHippocampal		33	-27	-18	5.04	160
R. Calcarine	18	15	-84	15	-6.90	144

**FIGURE 4:**
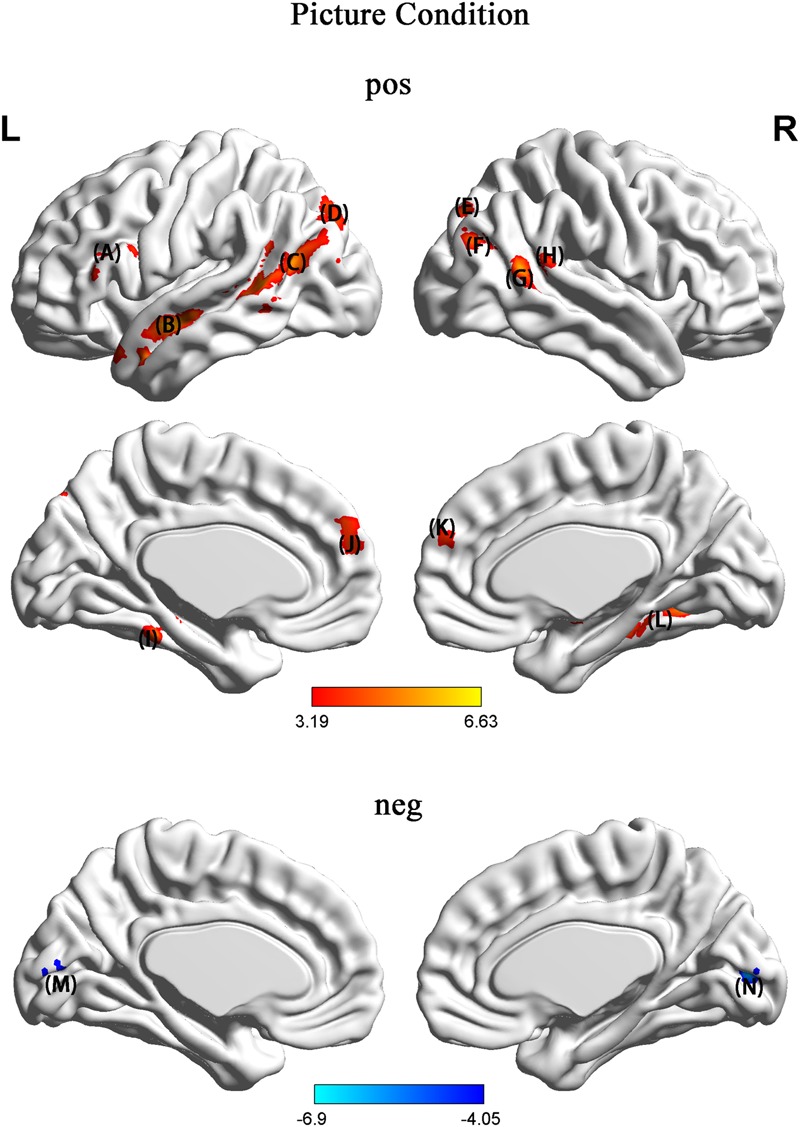
In contrast to NH, HU showed increased activation in regions such as the (A) left SFG and left triangle (IFG) (B) left MTG and STG (C) left TPJ and left fusiform gyrus (D) left SOG (E) right SOG (F) right MOG (G) right MTG (H) angular gyrus (I) left parahippocampal region (J) left prefrontal cortex (PFC) (K) right prefrontal cortex (PFC) (L) right parahippocampal region (M) left calcarine region (N) right calcarine region.

In the picture condition, in contrast to NH, HU showed increased activation in regions such as the left SFG, left triangle IFG, bilateral MTG, left STG, left fusiform gyrus, and parahippocampal and calcarine regions (**Figure 4** and **Table 2**).

#### The Common Regions between Character and Picture

**FIGURE 5:**
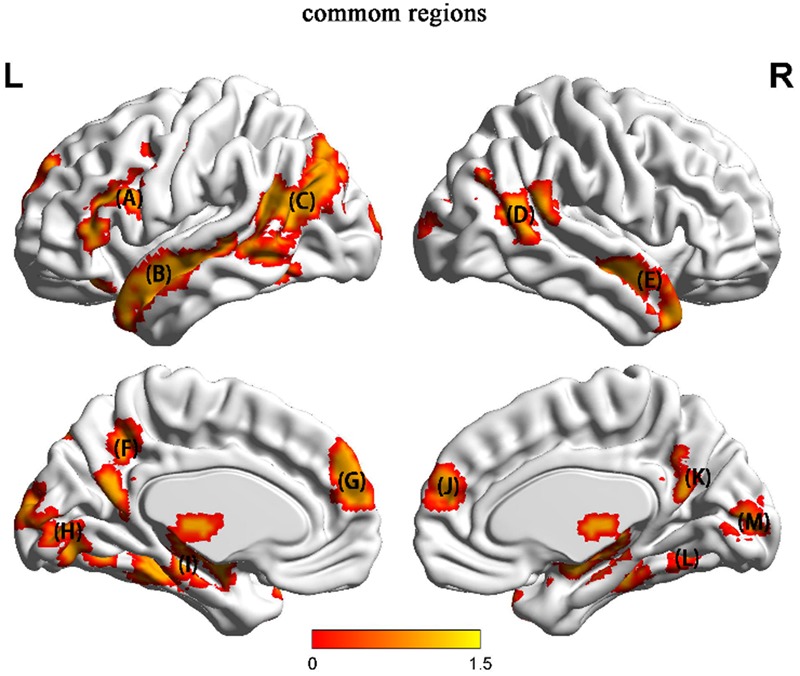
The common regions activated in both the character condition and the language-free condition. (A) left SFG and left triangle IFG (B) left MTG and STG (C) left TPJ and left fusiform gyrus (D) right MOG (E) right MTG (F) left SOG (G) left PFC (H) left MOG (I) hippocampus (J) right PFC (K) PCC (L) right parahippocampal region (M) right MOG.

Whole-brain analysis showed that certain regions were activated by both types of stimuli. Several of these regions were also identified in many of the studies previously reviewed. We conducted a conjunction analysis between the character (HU > NH contrast) and picture (HU > NH contrast) conditions to identify these common regions (**Figure 5**). The common regions were the left SFG, left triangle IFG, left TPJ, bilateral MTG, bilateral PFC and right parahippocampal region.

## Discussion

Humor comprehension and its neural mechanisms have been widely studied, yet no consistent conclusions have been drawn—for several reasons. First, previous studies have used different types of stimuli and problems involving multiple cognitive processes, such as incongruity detection, incongruity resolution and insight processing. Second, brain areas related to the character condition (verbal problem) are not directly comparable to brain areas related to the picture condition (visual problem) because only one type of problem (visual or verbal) has been used in previous separate studies. Third, many studies have not addressed the insight involved in humor comprehension and have not provided suitable conditions in their experiments. Therefore, the improvement provided by the current study was using two different types of experimental materials to study the neural basis of humor comprehension. Furthermore, we studied insight during humor comprehension.

The behavioral results were consistent with what we expected, indicating that the reliability and validity of our experimental materials were high. In addition, 79.0 ± 12.7% of the HU trials were rated humorous and surprising by the participants, indicating that the participants were likely surprised by the humorous ending and not surprised by the non-humorous ending. Among the participants, 75% said that they experienced insight when they read the jokes. We inferred that insight may be involved in humor comprehension. Our fMRI results indicated that in the character condition, in contrast to NH, HU showed increased activation in regions such as the left SFG, right MFG, left IFG, left precentral, bilateral MTG, right STG, left TPJ, bilateral MOG, PCC, sgACC, and hippocampus. In the picture condition, however, in contrast to NH, HU showed increased activation in regions such as the left SFG, left triangle IFG, bilateral MTG, left STG, left fusiform gyrus, and parahippocampal and calcarine regions. In line with the previous literature, we suggest that the MTG, SFG, IFG, and TPJ are involved in humor comprehension. As stated in our hypothesis, the key brain areas associated with humor comprehension are not affected by the types of experimental materials used.

The activation of the MTG has been observed in many studies related to incongruity detection (Cui et al., 2013), social signaling (Sugiura et al., 2013) and joke comprehension, particularly the right MTG in two (visual or verbal) conditions (Goel and Dolan, 2001; Moran et al., 2004; Chan et al., 2012; Shibata et al., 2014). In addition, the right MTG has been associated with semantic violations of language processing (Kuperberg et al., 2000; Ni et al., 2000; Newman et al., 2001). Lambon Ralph et al. (2010) suggested that the MTG is activated in detecting incongruity because it’s functions in recognizing and categorizing stimuli, and Chan and Lavallee (2015) reported that the MTG is associated with the process of bridging-inference joke comprehension. In light of previous results, we inferred that MTG is a key region involved in incongruity detection.

Recent studies have suggested that the left SFG might contribute to the connection of a joke’s setup to its punchline (Samson et al., 2009; Bekinschtein et al., 2011; Shibata et al., 2014); thus, it might play a key role in the integration of humor comprehension. The left SFG is also related to cognitive processes such as organizing ideas, obtaining insights, and successfully solving ambiguous sentences (Samson et al., 2008, 2009). The results of Samson et al. (2009) and Chan et al. (2012) suggest that the main reason for the activation of the left SFG is that incongruity resolution requires more coherence building, more mental manipulation and the re-organization of context. The left SFG functions in attempting to “make sense” or “make attribution” during humor comprehension (Samson et al., 2008). Furthermore, the left SFG contributes to improving cognitive functions, particularly working memory (Owen, 2000; Petrides, 2000). In the current study, in contrast to the NH, the HU required more executive processing and mental manipulation during humor comprehension.

The present study also revealed increased activation of the IFG in humor comprehension. Previous studies have found that the left IFG is involved in semantic comprehension, humor detection and the resolution of semantic ambiguities (Moran et al., 2004; Azim et al., 2005; Bekinschtein et al., 2011). Bilateral IFG also plays an important role in the construction of a situation model, the presentation of an ambiguous statement, exaggeration jokes, and ambiguity jokes (Ferstl et al., 2005; Rodd et al., 2005; Zempleni et al., 2007; Menenti et al., 2009), and these tasks are associated with executive control processes, such as language-based decoding and retrieval from episodic memory (Chan and Lavallee, 2015). One study discovered greater activation of the IFG in switching compared with self-reported clustering and free generation (Hirshorn and Thompson-Schill, 2006). In incongruity detection, the subjects of a separate study realized that there were two or more incompatible schemas (Wyer and Collins, 1992). Incompatible schemas entail cognitive disfluency and lead to immediate negative affect (Topolinski and Strack, 2015). When the punchline appears, the IFG subserves the switching mechanism; it can promote semantic fluency. Consequently, this fluency helps the core mechanism of incongruity resolution, thus increasing the funniness of a joke (Leavitt and Christenfeld, 2011; Topolinski, 2014). Therefore, we tended to believe that the IFG is crucial to language-related humor (Chan et al., 2012; Shibata et al., 2014).

The activation of the TPJ might be related to the process of inferring knowledge, the integration of multi-sensory information and coherence building (Gick and Lockhart, 1995; Ferstl and von Cramon, 2002). The TPJ has been associated with both high-level social-cognitive processes and low-level computational processes (e.g., attention orientation) (Decety and Lamm, 2007), and it is also a key region in insight studies (Starchenko et al., 2003; Bechtereva et al., 2004; Kounios et al., 2006). As previously mentioned, a series of studies reported that the TPJ is closely related to incongruity resolution during the semantic processing of jokes, the integration of large amounts of information and the funniness of a joke (Goel and Dolan, 2001; Mobbs et al., 2003; Samson et al., 2008, 2009; Amir et al., 2013). By generating, testing and correcting internal predictions about external sensory events, the TPJ helps make sense of an incongruity. Therefore, the activation of the TPJ in our study could be explained by its function in generating and integrating information.

Our fMRI experiment also allowed for the investigation of the insight element during humor comprehension. The results support our hypothesis that (a) the STG, MTG, IFG, and TPJ are activated in character (verbal) or language-free cartoon (visual) conditions simultaneously. These regions appear to be involved in incongruity detection and resolution; (b) the common brain regions activated in both verbal and visual conditions are the MTG, STG, cingulate gyrus, fusiform gyrus, and IFG. These regions appear to be critical regions in detection and resolution of the incongruity. These findings indicate that the insight moment experienced during humor comprehension is universal regardless of the type of stimulus.

We also observed the activation of the sACC in humor comprehension. Previous studies have shown that the sACC is related to negative emotions. For example, a review of brain imaging against the background of the four-region model showed significant activation of the sACC during sad events (Vogt et al., 2003). In addition, one of the first brain imaging studies discovered that the activation of the sACC subregion is associated with negatively valenced affect in fit women (George et al., 1995). In our study, when participants were confronted with incompatible schemas in humor detection, the unexpected events sometimes led to surprise (Wyer and Collins, 1992). Surprise could be regarded as an interruption mechanism (Meyer et al., 1997), and such interruptions not only affect one’s cognitive processes but also one’s mood (e.g., fear, sadness, and surprise) (Noordewier and Breugelmans, 2013). Moreover, one study discovered that higher corrugator activity is elicited by more surprising trivia compared with less surprising trivia, and higher corrugator activity indicates more mental effort and negative effect (Topolinski and Strack, 2015). Noordewier et al. (2016) also found that instant cognitive interruption triggers negative effect in the process of surprise. Hence, we believe that the activation of the sACC in humor comprehension might be related to negative feelings, which are produced when thought is interrupted by unexpected events.

Our results also showed the activation of brain regions often implicated in (non-humorous) insight tasks, such as the PFC, STG, and TPJ. According to our study, we could conclude that the type of (joke) stimuli we presented did function as a type of insight task. The activated brain regions observed in our study, such as the PFC, STG, and TPJ, are in agreement with those reported in previous studies about insight. We could infer that the task we presented in the experiment is similar to previous insight tasks. Luo and Niki (2003), Luo et al. (2004) explored the neural basis of insight by presenting a trigger (the solution) to catalyze the process of solving insight problems; the exercise was a passive insight task in which the resolution was presented rather than formulated by the participant (Dietrich and Kanso, 2010). The results indicate that certain regions, such as the PFC, MTG, posterior parietal cortex and hippocampus, are involved in insightful riddle-solving. Other researchers have discovered that the PFC contributes to conflict resolution of working memory, semantic selection and the shift in cognitive set (Thompson-Schill et al., 1997; Jonides et al., 1998; Monchi et al., 2001), while the function of the PFC in breaking a mental impasse (insight process) might be related to conflict resolution (Luo et al., 2004). Moreover, the involvement of the hippocampus in the insight process implies that a navigation-like process might occur during problem solving (Luo and Niki, 2003). EEG and fMRI studies have also indicated that insight moments are activated in the STG and TPJ, suggesting that the TPJ might play an important role in flexibility of thinking (e.g., switching and planning), formation of rich association and imagination, which might be related to the early stages of creativity (Starchenko et al., 2003; Kounios et al., 2006; Qiu et al., 2008a). These results are in line with our hypotheses suggesting that humor and insight share certain activated brain regions in common.

## Conclusion

To conclude, no consistent conclusions about the neural basis of humor comprehension and insight have been drawn for several reasons. First, most researchers have used only one stimulus in their study, preventing them directly comparing visual materials with verbal materials. Second, previous studies about humor and insight have treated the two topics separately although they have some similarities with respect to cognitive processing and neural mechanisms; indeed, no study has addressed both humor and insight. Therefore, using two different kinds of materials to study the insight during humor comprehension were the improvement of present study. The results of our behavioral experiment showed that the experimental materials pertaining to humor are reliable. The results of our fMRI experiment are consistent with our assumptions that the activation of different brain region is not affected by the type of material presented and that the brain regions we identified in humor comprehension overlap with those reported in previous studies about insight. However, our conclusions were drawn from a sample of only dozens of people by using visual materials and verbal materials. The conclusions must be confirmed in a larger sample in future studies to better understand the neural basis of humor comprehension.

## Author Contributions

FT and YH contributed significantly to analysis and manuscript preparation. QJ, QZ, GC, and AD contributed to the conception of the study. YH, WZ, and WY contributed to the fMRI data acquisition. QC and JS contributed to the fMRI data analysis.

## Conflict of Interest Statement

The authors declare that the research was conducted in the absence of any commercial or financial relationships that could be construed as a potential conflict of interest.

## Footnotes

1 http://www.fil.ion.ucl.ac.uk/spm/
